# Assessment of the Effect of Intestinal Permeability Probes (Lactulose And Mannitol) and Other Liquids on Digesta Residence Times in Various Segments of the Gut Determined by Wireless Motility Capsule: A Randomised Controlled Trial

**DOI:** 10.1371/journal.pone.0143690

**Published:** 2015-12-02

**Authors:** Ivana R. Sequeira, Roger G. Lentle, Marlena C. Kruger, Roger D. Hurst

**Affiliations:** 1 School of Food and Nutrition, Massey University, Private Bag 11222, Palmerston North, New Zealand; 2 The New Zealand Institute for Plant & Food Research Ltd, Palmerston North, New Zealand; The Lee Kong Chian School of Medicine, SINGAPORE

## Abstract

**Background:**

Whilst the use of the mannitol/lactulose test for intestinal permeability has been long established it is not known whether the doses of these sugars modify transit time Similarly it is not known whether substances such as aspirin that are known to increase intestinal permeability to lactulose and mannitol and those such as ascorbic acid which are stated to be beneficial to gastrointestinal health also influence intestinal transit time.

**Methods:**

Gastric and intestinal transit times were determined with a SmartPill following consumption of either a lactulose mannitol solution, a solution containing 600 mg aspirin, a solution containing 500 mg of ascorbic acid or an extract of blackcurrant, and compared by doubly repeated measures ANOVA with those following consumption of the same volume of a control in a cross-over study in six healthy female volunteers. The dominant frequencies of cyclic variations in gastric pressure recorded by the Smartpill were determined by fast Fourier transforms.

**Results:**

The gastric transit times of lactulose mannitol solutions, of aspirin solutions and of blackcurrant juice did not differ from those of the control. The gastric transit times of the ascorbic acid solutions were significantly shorter than those of the other solutions. There were no significant differences between the various solutions either in the total small intestinal or colonic transit times. The intraluminal pHs during the initial quartiles of the small intestinal transit times were lower than those in the succeeding quartiles. This pattern did not vary with the solution that was consumed. The power of the frequencies of cyclic variation in intragastric pressure recorded by the Smartpill declined exponentially with increase in frequency and did not peak at the reported physiological frequencies of gastric contractile activity.

**Conclusions:**

Whilst the segmental residence times were broadly similar to those using other methods, the high degree of variation between subjects generally precluded the identification of all but gross variation between treatments. The lack of any differences between treatments in either total small or large intestinal transit times indicates that the solutions administered in the lactulose mannitol test of permeability had no consistent influence on the temporal pattern of absorption. The negatively exponential profile and lack of any peaks in the frequency spectra of cyclic variation in gastric intraluminal pressure that were consistent with reported physiological frequencies of contractile activity profile suggests that the principal source of this variation is stochastic likely resulting from the effects of external events occasioned by normal daily activities on intra-abdominal pressure.

**Trial Registration:**

Australian New Zealand Clinical Trials Registry ACTRN12615000596505

## Introduction

The lactulose mannitol (LM) test is well established in the clinical investigation of intestinal permeability [1–3]. Latterly it has been demonstrated that the rates of absorption and excretion of the probe sugars vary significantly with time from dosage [4,5]. A body of evidence indicates that there are segmental differences in permeability of the mucosa to lactulose and mannitol [6]. However, little work has been done to directly assess whether the osmolarities of these two sugars or the presence of substances such as aspirin and ascorbic acid, which are used in a recently developed LM test that is adapted to assess gut health, influence gut segment transit times.

The lack of work in this field is largely due to the lack of suitable techniques. Several methods have been used to assess gut segment transit times notably radiographic techniques, dosage with metabolizable markers that can be detected in the breath tests [7] and gamma scintigraphy [8,9]. Thus gastric transit time can be determined indirectly by quantifying carbon dioxide in expired air after consumption of ^13^C labelled octanoic acid [10] and small intestinal transit time similarly from excretion of ^13^C labelled glycosyl ureides [11]. However these methods do not directly assess the physical passage of the gut contents [12]. Again, whilst the use of scintigraphy may be well suited to the sequential determination of small intestinal [13], large intestinal and whole gut [14] transit times of pharmaceuticals, the technique requires dosage with isotopes such as Technetium 99 m [13].

Recent developments in wireless telemetry have made it possible to assess compartmental and total residence times by identifying changes in ambient luminal pH and pressure recorded during the transit of a wireless motility capsule (WMC) [15]. These capsules have recently become commercially available (SmartPill Corporation, Buffalo, NY, USA), are approved for use in determination of these parameters by the American and European Neurogastroenterology and Motility Societies [15] and have minimal harmful effects [16]. The use of the WMC to check compartmental transit times in relation to passage of permeability probes is thus convenient and non-invasive [17].

Some technical difficulties remain, thus for example the WMC may empty relatively promptly from the stomach if ingested during the intermeal interval i.e. phase III of the migrating motor complex cycle (MMC) compared with that ingested during the postprandial period [18]. Hence the manufacturers advise that concurrent administration of a small amount of nutrient with the WMC may prevent this occurring. Given that no mechanisms operate within the human small or large intestine to selectively retain either solid or liquid matter [19], it is likely that the WMC will transit at a similar rate to other particulate matter and in synchrony with the liquid phase, especially during the inter meal interval. This hypothesis is supported by data showing strong correlation between the gastric emptying of a radiolabelled meal and that of the WMC [20] and between colonic transit times determined using radio opaque markers and those determined using the WMC [21]. Hence it appears that transit times derived directly from the transit of the WMC may be as clinically relevant as those derived indirectly by other clinically accepted methods [22].

The purposes of the current work were firstly to identify whether the doses of lactulose and mannitol that are used in the LM test of intestinal permeability had any effect on the transit times of a proprietary WMC, the SmartPill. Secondly to determine whether a 600 mg dose of soluble aspirin that is used to promote intestinal permeability in a recent modification of the LM test designed to assess gut health [4] had any effect on Smartpill transit times. Thirdly to determine whether ascorbic acid (a weak organic acid) or blackcurrant extract, substances that have been shown to promote gastrointestinal wellbeing; by ameliorating oxidative damage to the mucosa [23,24,25,26] and by influencing the growth of beneficial groups of microflora [27,28] respectively, had any effect on Smartpill transit times. These comparisons were necessary as any alteration in the timings of the peaks in absorption of the sugar probes could influence assessments of gut permeability.

## Method

### Ethical approval

Ethics approval was obtained for the study from the Massey University Human Ethics Committee: Southern A 12/42. Before they commenced the trial subjects were required to give written informed consent. Participants were recruited from October 2012 to February 2013. The trial was successfully registered with the Australian New Zealand Clinical Trials Registry (ANZCTR): ACTRN12615000596505.

### Selection and screening of subjects

**Fig 1:**
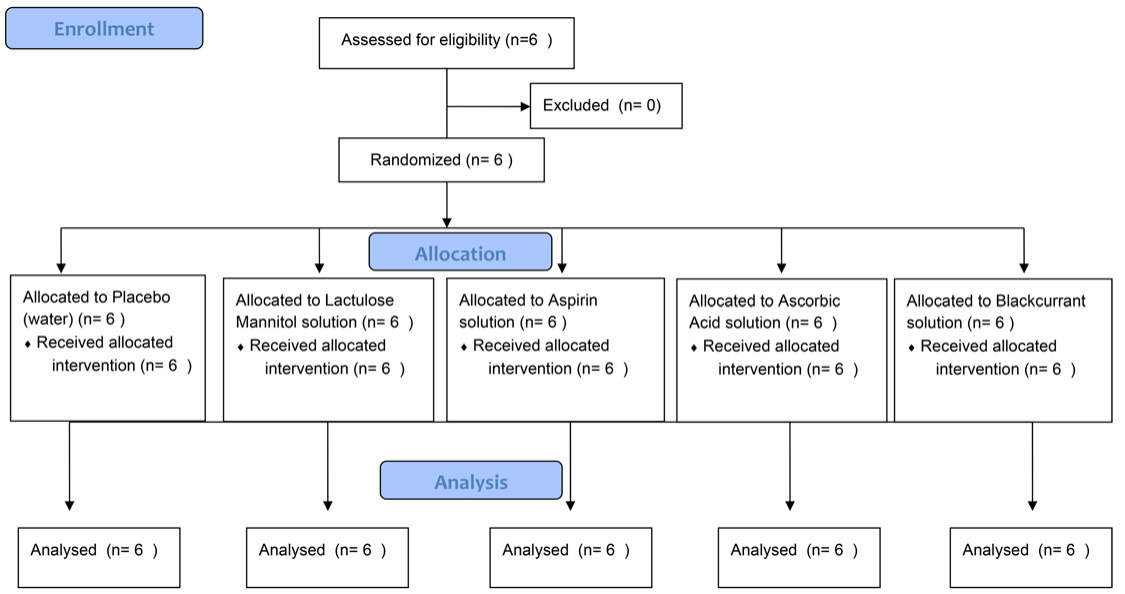
CONSORT flow diagram of the progress through each phase of the study.

Six healthy female participants between 20–40 years of age (mean age: 30 y) were recruited (Fig 1). Female participants were used as prior work in developing a more accurate methodology for the LM test [29] and work modifying the test to include dosage with an agent that increased intestinal permeability [4,5] had used only female participants. Further, that other work had identified significant variation in intestinal permeability with gender [30,31]. There is no data available regarding variation of gastric and intestinal motility and residence time using the SmartPill® and the drinks used in the expanded lactulose mannitol test. Hence data reporting variance between normal healthy subjects which could provide a meaningful test of statistical power were not available. Six subjects were chosen based on previous studies using the SmartPill® that have used a similar sample size and obtained statistically significant results [32,33].

Whilst the likelihood of chronic gastric retention of the SmartPill® is known to be low [15], we restricted participants to those of medium body size on the assumption that they would have a larger gastric volume and pyloric diameter [34]. Again subjects were restricted to those with a body mass index (BMI) of < 35 kg m^-1 2^ so as to avoid any attenuation of the WMC signal by overlying abdominal adipose tissue and interference with detection by the data logger [35]. Hence the recruited participants were 162–172 cm tall (mean: 167 cm) and weighed 60–73 kg (mean: 66 kg).

All subjects were screened with a health questionnaire to exclude those with a personal or strong family history of chronic gastrointestinal (GI) disorders or recent abdominal pain, nausea, vomiting, diarrhea, passage of blood and mucus in stools. Similarly subjects with a history of dysphagia, gastric bezoars, strictures, fistulas, bowel obstructions, diverticulitis, or previous GI surgery [33] were excluded. Subjects with an implanted electromechanical medical device, smokers, those with an intake of alcohol in excess of one standard drink per day, those with aspirin sensitivity and those taking regular prescription or over the counter medications (OTC) were also excluded. Similarly those subjects who consumed drugs that were likely to influence GI transit time, those consuming vitamins, prebiotics or probiotic supplements such as lactulose (S3 File). All participants were reviewed by a clinician to validate their medical history and their responses to the questionnaire before they were admitted to the study.

Subjects consumed self-selected diets during the study period but were asked to avoid consuming foods and fruits that contained high levels of ascorbic acid or anthocyanins (S4 File). They also were asked to avoid consuming NSAIDs or alcohol for three days prior to and during the study period [22,35] and to avoid performing any vigorous exercise during this time [32].

### Experimental protocol

Each participant received each of the five treatments in a randomized sequence at weekly intervals, the latter calculated from the day when the SmartPill was voided. The randomization procedure for the order in which each subject received the various treatments was generated by a computer program. On the evening prior to each experimental session, each participant was required to consume a plain meal that was low in fat and fibre and to subsequently fast overnight. Upon arrival at the Human Nutrition Unit (Massey University, Palmerston North, New Zealand) each participant was given 250 ml of water to drink immediately. Fifteen minutes later they were fitted with a data-logger which was attached to a lanyard and maintained in close proximity to the stomach. They then swallowed the activated and calibrated WMC with 250 ml of one of five treatment solutions that also contained 5 g of glucose. Hence the five treatment solutions were water (placebo); 10 g lactulose (Duphalac®, Solvay Pharmaceuticals, NSW, Australia) and 5 g mannitol (Sigma-Aldrich, St. Louis, MO, USA) mixture; 600 mg aspirin (Disprin®; Reckitt Benckiser Healthcare, UK), 500 mg ascorbic acid (Hawkins Watts, New Zealand) and a blackcurrant extract containing 1167 mg total anthocyanins (Just the Berries Ltd, New Zealand). A brief description of the drinks is detailed in Table 1.

**Table 1:** Formulation of the various treatment solutions.

Solution	Content	Amount (ml)	Osmolarity (mOsmol/L)	pH
Placebo	Water + 5 g glucose*	250	103	7.54
Lactulose mannitol	Water + 10 g lactulose + 5g mannitol + 5 g glucose*	250	372	6.59
Aspirin	Water + 600 mg soluble aspirin + 5 g glucose*	250	129	5.30
Ascorbic acid	Water + 500 mg Ascorbic acid + 5 g glucose*	250	113	3.45
Blackcurrant extract	Water + 1167 mg anthocyanin + 5 g glucose*	250	116	3.85

* = 5% glucose added to avoid prompt gastric emptying on advice of the Smartpill manufacturer.

Each participant was subsequently monitored over seven hours in the human nutrition suite [36]. The participants were not allowed to eat during the study period; however they consumed 200 ml water four hours after commencement of the session. Participants were provided with a standardized meal at the conclusion of the experimental session before their departure [37]. They were asked to keep the data logger/receiver near their abdomen at all times (S5 File). Hence during activities such as taking a shower, they were instructed to place it in a dry area, no less the 5 feet from them [35]. They were also required to carry documentation (S6 File) with them at all times which would inform any medical personnel that they had ingested the SmartPill. Each participant was asked to complete an activity diary noting the timing of meals, the passage of stools, and the occurrence of gastrointestinal symptoms such as pain/discomfort, nausea, vomiting etc. and to press the ‘event’ button on the data recorder to time mark each of these events. Participants were asked to attend the laboratory on subsequent days, after collecting (S7 File) and bringing with them all stools they had voided. Each stool was collected into a time labelled container and held for three minutes after voiding to allow any decrease in ambient temperature to be detected by the data logger. This procedure was repeated until the capsule had been voided and identified. The data logger was subsequently downloaded via the software provided by the manufacturer (MotiliGI software, Given Imaging Corp).

### Data processing and statistical analysis

**Fig 2:**
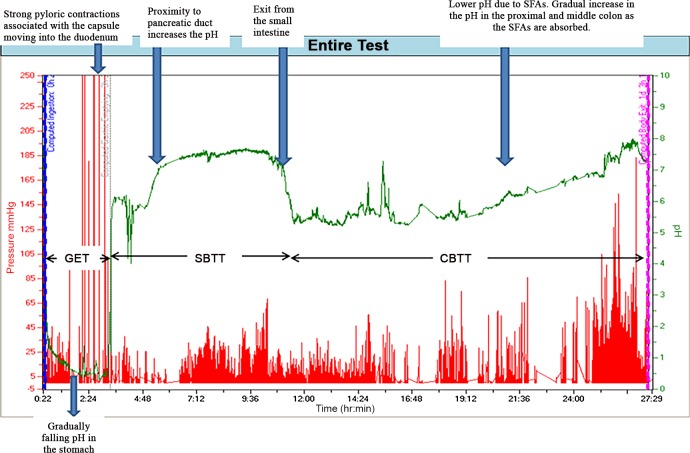
Record of variation in pH and pressure over time obtained by a wireless motility capsule in a healthy female after consumption of the placebo solution. The X axis represents time and the Y axis represents pH in green and pressure in red. Gastric emptying is indicated by a sharp rise in pH of > 4unit at 3 h and 21 m. Entry into the caecum is indicated by a drop in pH of 1 unit at approximately 10 h 35 m after the time of ingestion of the wireless motility capsule (WMC). The drop in temperature at 27 h and 24 m indicates the exit of the WMC. GET = Gastric emptying time; SBTT = Small bowel transit time; CBTT = colonic transit time; SCFA = short chain fatty acids.

Gastric emptying time (GET), small bowel transit time (SBTT) and colonic transit time (CBTT) were determined on a basis of change in pH, pressure and temperature profiles in a similar manner to that described by previous workers [15,32,33,36] (Fig 2).

Hence gastric residence times were determined as the interval from the time of ingestion of the capsule to that of a rapid rise in pH (> 3 pH units) [32] subsequent to the asymptotic intragastric decline in pH, that was synchronous with the time of cessation of pressure waves in excess of > 200 mmHg pressure. It is noteworthy that the retention times of the WMCs was likely to have been somewhat greater than the maximum residence times of the ingested liquid as residual particulate solids of greater than 1mm in linear dimension are known to exit the stomach after concurrently ingested liquid [18] on commencement of phase III of the MMC cycle [38,39].

The times at which the intragastric pHs stabilized after the initial exponential decline were determined by visual inspection of a plot of Log pH vs time (OriginLab, Northampton, MA). This enabled the half times (T½) of the exponential intragastric decline in pH for each of the five treatments to be determined. The mean T½ values following each of the five treatments in the six participants required transformation in the Johnson algorithm of the Minitab 16 statistical package [40] to render them amenable to parametric analysis. This software fits a functional transformation with outcome that best fits a normal distribution. Variation in transformed T½ values with treatment was assessed by repeated measures ANOVA.

Small intestinal residence times were taken from the time when the phasic generation of pressures in excess of 200 mmHg ceased to the time when there was a sustained fall in pH of at least 1.3 pH units [33] below the mean small intestinal pH i.e. pH > 4 [36]. This fall in pH was presumed to result from the production of short chain fatty acids (SCFA) from bulk fermentation of oligosaccharides by colonic microbiota [41,42]. The variation in pH during small intestinal transit was examined over four equal consecutive time periods, i.e. quartiles. The variations in pH during the four quartiles within each of the five treatments were compared by doubly repeated measures ANOVA.

Colonic transit times were determined from the time of the commencement of the fall in pH approximately by 1.3 pH units that marked the commencement of colonic fermentation until the time when the temperature dipped sharply below body temperature as a result of expulsion of the WMC [22,43]. The colonic trace was divided into two periods on the basis that the capsule was more likely to be in the proximal colon during the initial period and in the distal colon in the latter period. Hence differences in the pH levels during the two periods were compared by doubly repeated measures ANOVA.

We determined the range of frequencies in the pressure recordings during the time when the WMC was in the gastric lumen by fast Fourier Transform and then assessed the relative proportion of the frequency spectrum that was close to reported frequencies of antral contractions in the stomach, i.e. around 3 cycles per minute (cpm) [44,45]. The time series data for the pressure recordings were extracted from the MotiliGI software (Given Imaging Corp) and spectral density functions produced by Fourier transform in Matlab R2012a (The Mathworks, Inc., Massachusetts, US) for each participant following the consumption of the placebo.

Any data that was not normally distributed was transformed in the Johnson algorithm of the Minitab 16 statistical package [40]. This software fitted a function that best transformed the data set to one with a normal distribution. Statistical analyses were conducted in the SYSTAT statistical software package version 13 (Systat Software Inc., Chicago, IL) [46]. Differences in the transit times within segment following the consumption of aspirin, ascorbic acid, blackcurrant extract, lactulose mannitol solution and the placebo drink were assessed by repeated measures ANOVA of appropriately transformed data with the probabilities of *post hoc* comparisons corrected by the Bonferroni algorithm. In order to provide statistical statements that are suitable for inclusion in future comparisons degrees of freedom (d.f) and F values were included in all statements in addition to probabilities.

Covariance between the total transit times of the three major gut segments within subjects was also explored by multivariate analysis (principal component analyses) in the Minitab 16 statistical suite. Analyses were conducted using both the raw pooled data from the three segments and pooled data that were together transformed with the Johnson algorithm [47].

## Results

All six participants successfully completed the trial. No untoward gastrointestinal side effects, GI disturbances or retention of the pill were reported by any of the subjects during any of the experimental sessions following the intake of the SmartPill.

The gastric (-0.31 + 0.47*Asinh((X -1.29)/0.09), small bowel (0.22+0.57*Asinh((X-4.90)/0.32) and colonic (0.46+0.82*Ln((X+1.49)/(133.20-X)) total transit times of all six participants each required Johnson transformation to render them suitable for comparisons between treatments within segments by ANOVA. All gastric half-time data also required arcsine transformation (1.42+1.61*Asinh((X-0.38)/0.11) to render them amenable to ANOVA.

The quartile values for small intestinal pH also required arcsine transformation (1.93+0.73*Asinh((X-0.73)/0.08) to render them amenable to ANOVA.

All colonic pH data for the proximal and distal halves of colonic residence time were normally distributed and amenable to ANOVA.

The raw pooled untransformed transit times for each segment from each subject were used for multivariate analysis as well as the pooled, then Johnson transformed, data (1.03+0.46*Ln((X-0.43)/(117.24-X)).

### Stomach

**Fig 3:**
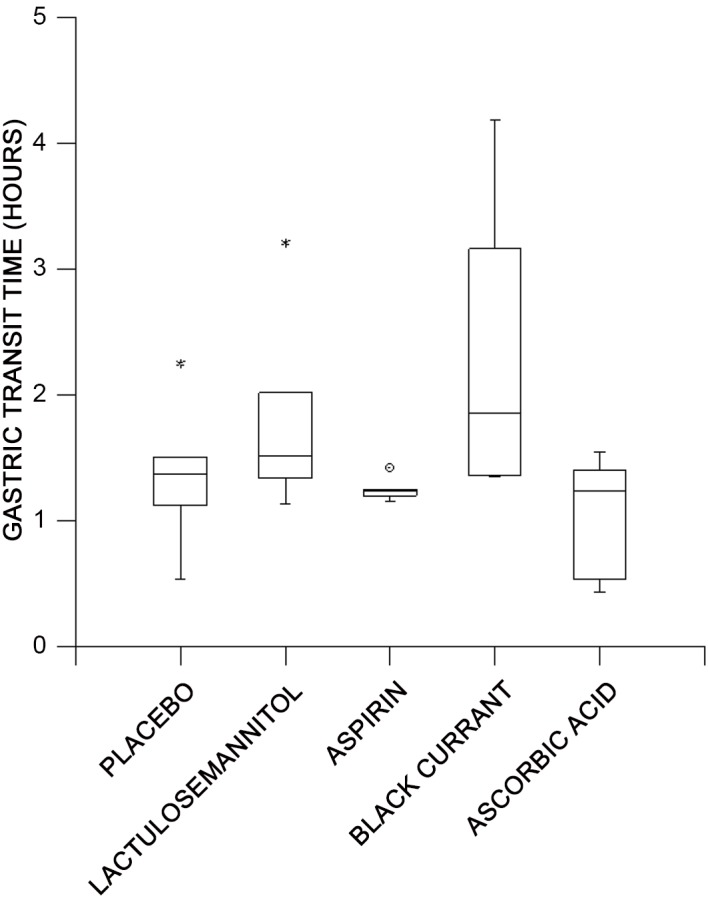
Variation with treatment in overall gastric emptying times determined by the wireless motility capsule in six healthy females. There were no significant differences in gastric emptying times between placebo (glucose solution) and solutions containing either lactulose and mannitol, aspirin or blackcurrant juice with the transformed data. However the emptying of the solution containing ascorbic acid was significantly slower (P = 0.005) on ANOVA than that containing lactulose and mannitol. * indicates the outlier in the pH data after the consumption of the placebo drink.

The overall mean gastric emptying time taken over all subjects for all treatments was 1.72 ± 0.2 h with large variation between subjects. Hence analysis of transformed values showed that the overall gastric emptying times for the placebo drinks (plain glucose solution) (mean: 1.36 ± 0.23 h), did not differ significantly from those containing glucose, lactulose and mannitol (mean: 1.79 ± 0.31 h), those containing aspirin and glucose (2.08 ± 0.83 h) or blackcurrant juice and glucose (mean: 2.30 ± 0.47 h) (Fig 3). However the transformed overall gastric emptying times for the ascorbic acid and glucose drink (mean: 1.07 ± 0.19 h) were significantly shorter (d.f 1,4; F = 30.88; P = 0.005) than those following consumption of solutions containing glucose, lactulose and mannitol.

The transformed half times based on the exponential decline in intragastric pH up to the point of stabilization differed significantly between the various treatments (d.f 4,20; F = 4.57; P = 0.009). Hence the gastric half times for the placebo drink were significantly shorter (mean: 0.16 ± 0.03 h) than those for either the aspirin drink (mean: 0.31 ± 0.04 h) (d.f 1,5; F = 10.21; P < 0.05), the lactulose mannitol drink (mean: 0.33 ± 0.04 h) (d.f 1,5; F = 12.59; P < 0.05) or the blackcurrant drink (mean: 0.32 ± 0.03 h) (d.f 1,5; F = 11.31; P < 0.05). Again, the half time for the ascorbic acid drink (mean: 0.22 ± 0.03 h) did not differ significantly from that for the placebo drink but was significantly lower than that for the aspirin (d.f 1,5; F = 6.9; P = 0.05) and blackcurrant drinks (d.f 1,5; F = 6.25; P = 0.05).

**Fig 4:**
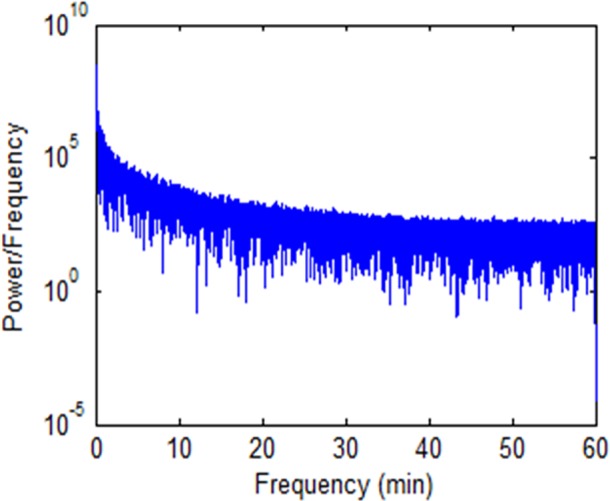
Spectral density of a fast Fourier transform (FFT) of variation in gastric pressure determined by wireless motility capsule in a single healthy female after consumption of the placebo solution.

The spectral densities of the pressure recordings obtained when the WMC was in the stomach, following the consumption of the placebo solution, were all negative exponentials with no peaks visible at or around the reported frequencies of antral contractions (Fig 4).

### Small intestine

**Fig 5:**
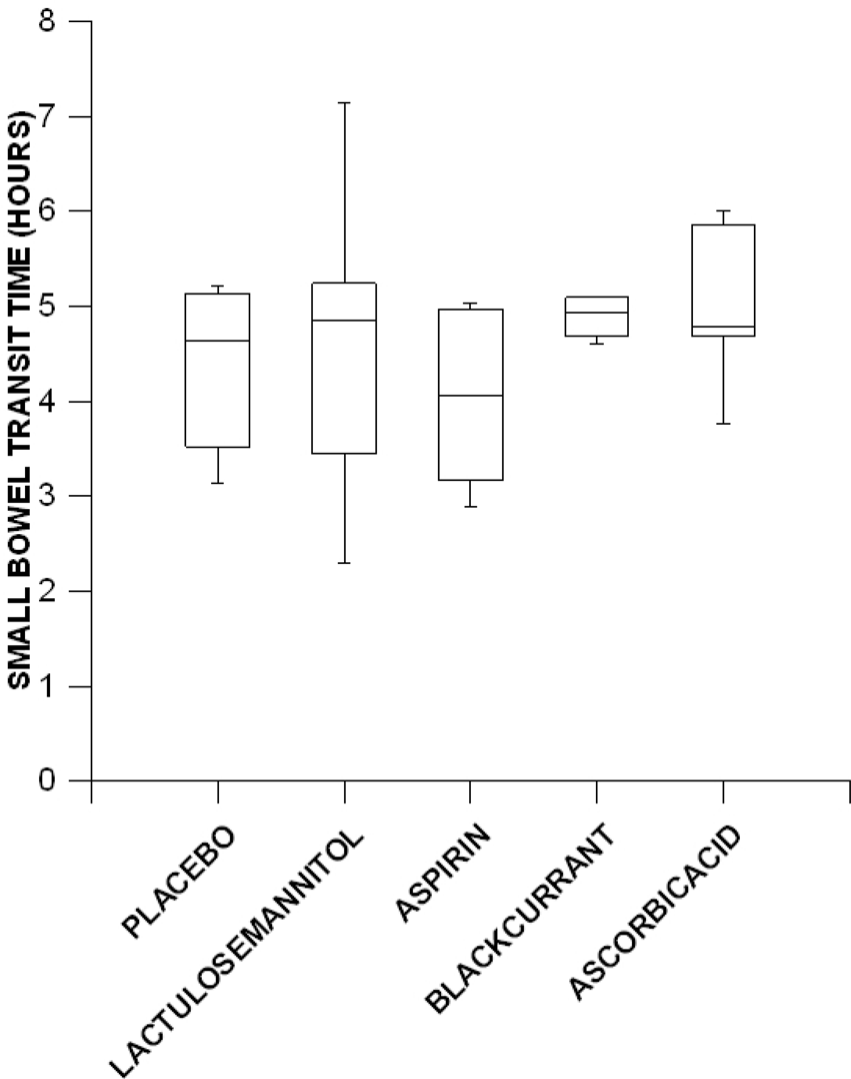
Variation with treatment in small bowel emptying times determined by wireless motility capsule in six healthy females.

The overall mean small intestinal emptying time for all subjects over all treatments was 4.63 ± 0.22 h but there was large variation between subjects. Analysis of transformed values showed that there were no significant differences between the overall small intestinal transit times for the placebo solution (mean: 4.38 ± 0.35 h), and those for either the lactulose mannitol solution (mean: 4.64 ± 0.68 h), the aspirin solution (mean: 4.03 ± 0.36 h), the blackcurrant solution (mean: 5.12 ± 0.63 h) or the ascorbic acid solution (mean: 4.98 ± 0.34 h) (Fig 5).

The ambient pHs during successive quartiles of the total time taken for the SmartPill to traverse the small intestine following consumption of the placebo solution differed significantly (d.f 3,12; F = 12.67; P <0.001). Hence, the pH levels during the first quartile were lower than those in the subsequent quartiles (Table 2) and generally rose progressively in succeeding quartiles. Similarly the ambient pHs during successive quartiles for the lactulose mannitol solution (d.f 3,12; F = 23.18; P < 0.05), the ascorbic acid solution (d.f 3,12; F = 28.62; P < 0.001) and the blackcurrant drink (d.f 3,12; F = 5.61; P < 0.05) all differed significantly, with the patterns of difference generally similar to those for the placebo a with progressive rise in pH over the first two quartiles (Table 2).

**Table 2:** Variation in lumen pH during four successive quarters of the time of transit through the small intestine after consumption of various drinks.

Treatment	Q1	Q2	Q3	Q4
Placebo	5.23 ± 0.27♦	6.61 ± 0.15●	7.35 ± 0.10■	7.48 ± 0.04▲	
LM	4.49 ± 0.65♦	6.60 ± 0.15 ●	7.42 ± 0.05■	7.52 ± 0.06▲	
BC	5.37 ± 0.25♦	6.85 ± 0.19♦	7.19 ± 0.08●	7.18 ± 0.09●	
AA	4.96 ± 0.27♦	6.37 ± 0.13♦	7.21 ± 0.10●	7.33 ±0.06■	
Aspirin	5.19 ± 0.21♦	6.80 ± 0.10♦	7.34 ± 0.07♦	7.50 ± 0.06♦	
Overall	5.06 ± 0.17♦	6.64 ± 0.07●	7.30 ± 0.04■	7.40 ± 0.04▲	

Results expressed as Mean ± SEM.

LM = lactulose mannitol BC = blackcurrant extract AA = ascorbic acid.

Q1, Q2, Q3, Q4 = quartiles of the small intestinal residence time.

Differences between the symbols (●, ♦, ■, ▲) between quartiles, for each treatment, denote that the means are significantly different (P < 0.05) from each other.

### Colon

**Fig 6:**
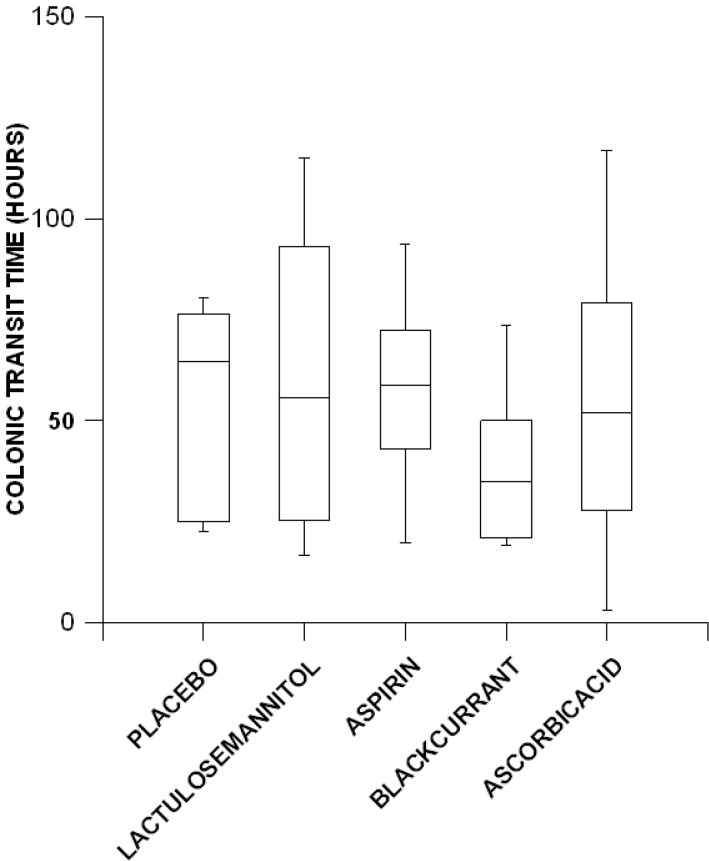
Variation with treatment in colonic emptying times determined by the wireless motility capsule in six healthy females.

The mean overall colonic transit time was 53.6 ± 5.58 h. The overall colonic transit times following the consumption of the placebo solution (mean: 55.67 ± 10.61 h) did not differ significantly from those for either lactulose mannitol solution (mean: 60.27 ± 15.80 h), the aspirin solution (mean: 57.81 ± 10.57 h), the blackcurrant solution (mean: 39.03 ± 8.71 h) or the ascorbic acid solution (mean: 55.16 ± 17.25 h) (Fig 6).

The overall pH levels in the initial half of the total colonic residence time were significantly lower (d.f 1,5; F = 11.81; P < 0.05) than those during the latter half (Table 3). There were no significant differences with treatment in the patterns of mean pHs during the initial and the subsequent half of the total colonic residence (Table 3).

**Table 3:** Variation in pH during two consecutive halfs of the time taken for the SmartPill to transit the colon after the consumption of various drinks.

Treatment	Initial period (proximal colon)		Latter period (distal colon)
Placebo	6.22 ± 0.24	7.20 ± 0.09	
LM	6.12 ± 0.14	7.36 ± 0.27	
BC	6.20 ± 0.28	7.08 ± 0.14	
AA	5.98 ± 0.17	7.26 ± 0.34	
Aspirin	6.35 ± 0.37	7.32 ± 0.14	
Overall	6.17 ± 0.11♦	7.25 ± 0.09●	

Results expressed as Mean ± SEM.

LM = lactulose mannitol BC = blackcurrant extract AA = ascorbic acid.

Differences between the symbols (♦, ●), denote that the mean pH is significantly different (P < 0.05) between the proximal and distal colon.

### Multivariate comparison of segment residence times

The Kaiser-Meyer-Olkin measure of sampling adequacy was 0.48. Using raw data the first component, PC1, represented 43% of the variance in the residence time of the three components of the gut, with successive components representing 33.3% and 23% respectively. Retention of the first 2 components yielded a model that accounted for 76.3% of variance (Table 4). The weightings of the first component indicated the total residence times in the large intestine (-0.71) were inversely correlated with those in the stomach (0.47) and small intestine (0.53). Similar results were obtained when pooled then transformed data were used.

**Table 4:** Principle component analysis of gastric and intestinal transit times.

Component	Initial Eigenvalues			Weighting of 1^st^ Component	%
	Total	% of Variance	Cumulative %		
1	1.292	43.064	43.064	GET	47
2	0.999	33.31	76.374	SBTT	53
3	0.709	23.626	100	CTT	-71

## Discussion

Broadly speaking, the determination of compartment residence times from changes in pH appears to give values that fit in with reports based on other methods of determination [13] [48]. The lack of any significant variation in the overall gastric, small intestinal and large intestinal transit times obtained with the different drinks indicated that none of their components greatly influenced the passage of digesta through the various segments. Moreover the mean gastric transit time of the placebo solution (1.36 ± 0.23 h) in the present study was close to that reported in another study using the SmartPill (1.53 ± 0.73 h)[18] in which 200 ml radiolabelled water was given to fasted subjects. The volume of water given in the latter study was reported to be insufficient to halt periodic phase III MMC induced contractions [18]. Hence it is likely that the glucose that was added to each drink in our study did not abolish phase III of the MMC cycle. Apart from the ascorbic acid drink; there were no differences between the gastric residence times of the various drinks used in our study. In this respect it is noteworthy that the ascorbic acid drink had the lowest pH (Table 1) and induced the lowest intra gastric pH. Given that the lactulose and mannitol solution contained the same concentration of the two sugars that are used in the improved Lactulose Mannitol test for intestinal permeability [4,5], it is evident that the dose levels of the sugars will not influence transit times.

The mean small bowel transit times over all treatments was 4.63 h, a value that is well within the overall range (3–6 h) reported by other workers [48] for healthy individuals, as was that of the lactulose mannitol solution (4.64 ± 0.68 h). Both values were close to the mean transit time determined on meta-analysis [13] of 201 gamma scintigraphic assays of the passage of various pharmaceutical dosage forms in healthy human subjects. The lack of any significant differences between the overall transit times of the various solutions through the small intestine indicates that lactulose and mannitol did not alter the transit of digesta through the SI during phase III of the MMC cycle. Hence, whilst the administration of lactulose has been reported to accelerate small intestinal transit [49,50], this did not occur with the concentrations that were used in the study and thus would be unlikely to occur with the standardized test of intestinal permeability [4,5].

The overall pattern of rise in ambient pH in consecutive quartiles of total small intestinal transit time following ingestion of the placebo fits in with reports that the capacity for the buffering in chyme increases distally along the small intestine [51]. Hence the lower pH in the proximal small intestine likely resulted from chyme having recently exited the stomach; whilst the higher pHs in the later quartiles presumably result from admixture with alkaline pancreatic and brush border secretions. This pattern of increase in intraluminal pH, and hence of buffering, appears robust as similar patterns of pH were obtained with all five drinks.

The mean overall colonic transit time (53.59 h) was within the range reported for healthy individuals, i.e. between 24–60 h [48]. The differences in lumen pH during the initial half of the total colonic transit time from those in the later half supports the hypothesis that increasing absorption of SCFAs generated by fermentation progressively lowers the pH of the colonic content [41,42].

The spectral density profile of pressure recordings taken whilst the WMC was in the cavity of the stomach, did not peak at or around the known frequencies of antral slow wave associated contractions [44,45]. The regular exponential form of the spectral density curve that was found in this study is thus likely to result from a series of random fluctuations in extraneous pressure over a wide range of frequencies rather than physiological events associated with the lumen. Hence the use of the motility index to detect peristalsis (calculated from the software as log_e_(sum of amplitude x number of contractions + 1)/time) may be inappropriate in ambulant or mobile subjects. We suggest that the use of the capsule to detect and analyse gastrointestinal motility may lack fidelity and needs further investigation [52].

The similarity of the gastric residence time to that reported during phase III MMC [18] indicates that the dose of glucose was insufficient to terminate this activity. Hence, under the conditions of the standardized lactulose mannitol test [4,5,29], none of the agents in the drinks is likely to influence transit time through a particular segment of gut. The finding also raises questions as to whether glucose-mediated negative feedback to gastric and other on flow [53] operates efficiently during phase III MMC.

Together the data indicate that the administration of the solutions in the expanded lactulose mannitol test does not change the temporal characteristics of transit through any particular segment. Hence, it is likely that the administration of any of these solutions will not result in significant delay or hurry of the passage of chyme and thus will not displace the peak time of excretion so as to influence the absorption of lactulose or mannitol at a given time after dosage.

## Supporting Information

S1 File CONSORT 2010 Checklist. (DOC)

S2 File Study protocol submitted to Massey University Human Ethics Committee. (PDF)

S3 File Information sheet given to participants. (PDF)

S4 File Food Avoidance list. (PDF)

S5 File Instruction sheet and guidelines for use of the Smartpill data logger. (PDF)

S6 File Documentation/Card. (PDF)

S7 File SOP for fecal sample collection. (PDF)

S8 File Data Set. (DOCX)
