# Supplementary material for: Assessment of the Effect of Intestinal Permeability Probes (Lactulose And Mannitol) and Other Liquids on Digesta Residence Times in Various Segments of the Gut Determined by Wireless Motility Capsule: A Randomised Controlled Trial
Source: PLoS One. 2015 Dec 2;10(12):e0143690. doi: 10.1371/journal.pone.0143690 (PMC4667890; doi:10.1371/journal.pone.0143690)
Supplement: S2 File — (PDF) [file pone.0143690.s002.pdf]

Study protocol submitted to the Massey University Human Ethics Committee (MUHEC) as part of the application form.

12. Outline the research procedures to be used, including approach/procedures for collecting data. Use a flow chart if necessary.

Potential participants who express interest in the study will be given an information sheet (attached), instruction sheet for use of the SmartPill and general guidelines (attached), SOP for stool collection (attached), food avoidance list (attached) and a consent form (attached).

6 healthy female participants of medium to large body size (165-170cm; 65-70kg) between the ages of 20-40 years will be recruited following completion of a Health Screening Questionnaire that is designed to exclude persons who have had any form of medical history or surgical procedure or who are consuming any medication that may interfere with the normal passage of the device through their GI tract. Only participants who meet the requirements of the study and have given written consent will be recruited.

Each participant will also be asked to avoid consuming foods such as berry and citrus fruits during the day prior to and on the day of the study. They will each be given an information sheet detailing these and other foods they will need to avoid (attached to application). Similarly each participant will be asked to refrain from taking OTC medication (with the exception of contraceptive medication) for at least one week prior to the test and to refrain from alcohol for at least three days prior to and during the course of the study.

On the evening prior to the experimental session each participant will be required to have a meal that is low in fat and fibre and not spicy so as to avoid GI irritation the details of which are given in the food avoidance list. Subjects will be asked to refrain from consuming any food or drink from 10.00pm the night before the study and be requested to attend the Human Laboratory at IFNHH the following morning at 8.00am. On each day that the participants are scheduled to attend their experimental sessions:

- Immediately after arrival the subjects will be asked to put on the data logger/receiver (on a lanyard) whose function will have already been checked to receive and record data from the Smartpill (calibrated with the buffer solution provided) that the subject is about to receive. The subject will then be asked to consume 400ml of the treatment solution (chilled to 0 centrigrade - this allow us to time mark the arrival of the liquid in the stomach). Participants will each receive 600mg soluble aspirin, vitamin C 500mg, berry drink, water or the test solution containing 10g lactulose and 5g mannitol at weekly intervals. The order of the treatments will be randomized. Five minutes later they will be required to swallow the capsule with 100ml of lukewarm distilled water.
- They will subsequently be monitored during the 8 hours they are at the laboratory. Participants will be allowed to eat a standardized meal that we will provide at the end of the experimental session and thereafter resume their normal diet.
- Subjects will be instructed to contact the researcher by telephone provided should they have any difficulty if they experience any discomfort during the period it takes to pass the capsule (Typically, healthy participants should void the capsule within 3 days, however in some individuals this could take up to 5 days). Additionally, arrangements will be made for the researcher to contact them by phone daily at a predetermined time to assess their well being.
- Subjects will be asked to strictly adhere to the instruction sheet and guidelines provided for the use of the SmartPill® receiver (attached) so that they continue to log data onto the receiver at all times.
- Subjects will be instructed to follow the SOP for stool collection that each of them will be given (attached) and to collect each stool in one of the plastic bags provided, and to label it with the date and time. These bags are designed to fit over the rim of the lavatory seat and the plastic bowl (which will be placed onto the seat) that we will provide them with (details inserted in the SOP).

- Additionally, subjects will be instructed when they have bowel movement to leave in the bag on the seat and within the toilet for 3 minutes; the time allowing for the SmartPill® receiver to detect a temperature decrease, indicating the SmartPill® has been passed. They will also be asked to briefly look at the stool to visually confirm the passage of the capsule. In the absence of such confirmation they will be asked to continue to collect subsequent stools in the manner described (detail in the information sheet)
- Each subject will be also given will be given a large biohazard bag in which to place all stool collection bags for subsequent delivery to the Human Nutrition Unit at IFNHH.
- Hence they will be instructed approximately 48-120hours after ingestion of the capsule, to revisit the Human Nutrition Laboratory and return the data receiver.

Kindly note: this has been extracted as part of the MUHEC application form.

FLOW CHART FOR RECRUITMENT & RANDOMIZATION:

Recruitment: **Advertisements** (placed in local newspapers and Massey University Campus advertising)

↓

Phone response from potential participants

↓

Information sheet + instruction and general guidelines for SmartPill use + SOP for stool collection + Food avoidance list + Consent form sent

↓

Potential participants reply

↓

Screening of participants by ***Health Screening Questionnaire***

↓ **Inclusion** | ↓ Exclusion

↓

Written signed **consent form collected**

↓

****Randomized** to receive each of the five treatments a week apart*:

- a negative control (water)
- lactulose mannitol test solution
- ascorbic acid 500mg
- aspirin 600mg.
- berry extract drink

*After they pass out the capsule.
