# Supplementary material for: Assessment of the Effect of Intestinal Permeability Probes (Lactulose And Mannitol) and Other Liquids on Digesta Residence Times in Various Segments of the Gut Determined by Wireless Motility Capsule: A Randomised Controlled Trial
Source: PLoS One. 2015 Dec 2;10(12):e0143690. doi: 10.1371/journal.pone.0143690 (PMC4667890; doi:10.1371/journal.pone.0143690)
Supplement: S3 File — (PDF) [file pone.0143690.s003.pdf]

An assessment of the effect of food/neutraceuticals on transit time through various components of the human gut

I would like to invite you to participate in our study. Below is important information to tell you more about our research. Thank you for your interest.

INFORMATION SHEET

Who are we?
I am Ivana Sequeira undertaking research in the Digesta Group for my PhD studying gut health and wellbeing. Our group is part of The Institute of Food, Nutrition and Human Health, Massey University. My project is investigating the effect of neutraceuticals on gut motility and transit time.

Why are we doing this trial?
Our group is studying the effect that molecules/nutrients have on the passage of food along the gut and the absorption of nutrients by the intestine. The current study investigates the rate at which different drinks flow through the different compartments of the gut and their effect on gut contractions. We will do this using a wireless motility capsule called the SmartPill® (SmartPill®; SmartPill Corporation, Buffalo, NY, USA). This capsule is swallowed and as it traverses the gut it records details about transit and gut contractions and transmits them to a data logger that is to be worn by you at all times. The capsule is 11.7mm x 26.8mm in size and you will need to swallow one after each solution has been consumed i.e. five times over five different days, a week apart from when the capsule has been passed out. Each capsule will be used only once.

How much time will you spend on the trial?
If you are keen on participating you will be asked to attend the laboratory on **six occasions**. Your first visit will be a *screening session* where your height and weight will be recorded –
you will be asked to complete a health questionnaire for us to assess your suitability to participate in the study. We will familiarise you with the data receiver and the capsule that you will receive. We will also demonstrate the standard operating procedure that you will be asked to follow whilst collecting stool samples. Please do not hesitate to ask us any questions that you may have.

If eligible to participate in the study, you will then be asked to attend our laboratory on ***five occasions; each session one week apart after you have passed out the SmartPill®***. On each occasion you will be given **either** of the following drinks:
- a soluble aspirin drink (600mg)
- vitamin C (500mg)
- water (100ml)
- berry drink
- lactulose mannitol solution

Subsequently, you will be required to stay at the human nutrition unit for the next eight hours so that you can be monitored. Videos and books will be available. Feel free to bring in your own reading material/study material, laptops etc (you will be able to access the Wifi connection). This study will take 43 hours of your time i.e. 8.5 hours each day to come to the laboratory.
We will give you a meal on each day when the experimental session is completed and compensate you for your time after each experimental session.

As we require you to confirm that you have passed out the capsule, every time you have a bowel motion, you will be asked to collect it in a plastic bag (provided) that is placed within the lavatory (kindly thoroughly read the standard operating procedures given to you for more detail) over two to three days until the capsule has been passed. *Once you have passed out the capsule you will not be required to collect any subsequent stool samples.* You will be provided with rubber gloves to handle the bags and a sealable storage biohazard bag to keep the samples in pending delivery to our laboratory at the time you will come in to hand over the data receiver to us. You will be required to fill out a diary that we will provide you with so that we have a record of the time as well as the 'event'. An 'event' is a meal that you have ingested, any drink you might have, a bowel movement etc.
Kindly note you are NOT to perform any vigorous or aerobic activity a day before and during the time when you have ingested the capsule.

Would you like to participate?
We would like to invite women, aged between 20 and 40 years to join our study. To be suitable to join in our study you should NOT:

- Be of small stature i.e. less than 165cm; less than 65 kg
- Be a smokers or tobacco chewer
- Consume more than one standard alcohol drink per day (6 glasses of wine, 3 pints of beer or 4 nips of spirits per week)
- Have a history of gastrointestinal diseases including a recent gut infection evidenced by a recent history of abdominal pain, nausea, vomiting, diarrhea, passage of blood and mucus in stools.
- Have had recent immunisation e.g. flu vaccine in the last month
- Have had recent major surgery e.g. abdominal surgery in the last three months
- Suffer from any inter current gastrointestinal disorder (contraindicated dysphagia (difficulty in swallowing), gastric bezoars (hair ball), strictures, fistulas (hole in your abdominal wall), bowel obstructions, diverticulitis, previous gastrointestinal surgery that could potentially cause capsule retention in the stomach (as has rarely been reported).
- Have implanted electromechanical medical devices and medications that influence gastrointestinal transit time.
- Be taking any ongoing prescription or over the counter medication or multivitamins except for contraceptive pills
- Have an earlier diagnosis of diabetes, cancer, Crohn's disease, ulcerative colitis, irritable bowel disease.
- Have a BMI < 18.5 or $> 30 kg/m^2$
- Be pregnant or lactating
- Have irregular menstrual cycles
- Have a high dietary fibre intake or be on a vegetarian diet
- Have food allergies.
- Have a history of or current urinary tract infections, vaginal conditions that cause discharge
- Have *aspirin* sensitivity
- Take ongoing prescription medication, antibiotics, over the counter medication (OTC) or multivitamins.

- Take prebiotic, probiotic and fibre supplements such as lactulose.

What are we going to measure?

Medical assessment**:**

We will ask you to fill in a form about your health and current medications.

SmartPill® intake:

The wireless motility capsule (SmartPill®; SmartPill Corporation, Buffalo, NY, USA) is shaped like any ordinary medicine capsule, measures 11.7mm x 26.8mm in size and has sensors for pH, pressure and temperature. If you have doubts about whether you can swallow the capsule we will give you a dummy capsule that you can swallow. When swallowed, the SmartPill® captures biomedical data from within the entire GI tract and wirelessly transmits it to a small receiver worn on a belt clip by you. The SmartPill® records changes in pH, pressure and temperature as the capsule traverses the gut lumen enabling gastric emptying time (GET), small bowel transit time (SBTT), colonic transit time (CTT) and whole gut transit time (WGTT) to be accurately determined.

Protocol:

You will be instructed to commence the study at 8:00am (5 sessions a week apart from when you have passed out the capsule) at the Human Nutrition Unit and will have to avoid consuming foods such as berry and citrus fruits that have been detailed in the Food Avoidance List a day before the start of the study. Additionally you will be asked to refrain from taking OTC medication (with the exception of contraceptive medication) for at least one week prior to the test and to refrain from alcohol at least three days prior to the test and during the course of the study.

On your arrival at the laboratory following an overnight fast (10-12 hours i.e. your dinner needs to be eaten by 8.00pm the previous night),

- You will be fitted with the SmartPill® receiver which is attached to a lanyard that goes around your neck (please read thoroughly the guidelines and instructions for the use of the receiver; importantly, keep the data receiver on your body at all times whenever possible throughout the test; DO NOT wear it whilst bathing and showering BUT hang it in the bathroom near you in a position where it will not get wet, the receiver needs to be kept dry and as close to the body as possible).
- You will then receive a 400ml drink of either *600mg soluble aspirin; vitamin C (500mg), water, berry drink or lactulose mannitol solution* on separate occasions a week apart.
- 5 minutes later you will then be required to swallow the SmartPill® with 100ml of water.
- For the next eight hours you will be required to stay at the Human Nutrition Unit so that we can monitor you (data will be recorded via the receiver worn by you).
- You will not be allowed to eat during the collection period but will be asked to drink a further 200ml water at three hours after the start of the session.
- We will provide you with a standardized meal after the eight hour period.
- Thereafter you can resume your normal diet (avoid alcohol).
- You will have to record and describe any 'events' in your diary i.e. the time you have a meal or a drink, have a bowel movement etc.
- Kindly read the standard operating procedure for stool collection that is provided as every time you need to go to the toilet you *will have to follow the protocol.*
- Every time you have a bowel movement you must first insert the plastic bowl provided into the bowl of the toilet and place the collecting bag onto it with the sides of the bag drawn up over the toilet seat. Let the stool remain in the toilet for 3 minutes; this time allows for the SmartPill® receiver to detect a temperature decrease, so that it can record when the

SmartPill® has been passed out of your body. Additionally you will be asked to visually confirm the passage of the capsule. Kindly record this in your diary.

- If you experience any difficulty or discomfort kindly do contact us on the numbers provided at the end of the information sheet.
- You will revisit the Human Nutrition Laboratory approximately 48-120hours after ingestion of the capsule, and return the data receiver along with the biohazard bag (containing all your stool sample bags) that we have provided you with.

Thus, you will attend the laboratory on five occasions a week apart where you will receive the drinks/treatments in a randomised sequence. The total amount of time you will spend in the laboratory is 43 hours.

Are any of the procedures harmful or painful?
This procedure is routinely used as a clinical test and should not cause any discomfort. On rare occasions the passage of the capsule may be delayed. In order to detect any such delay you will be monitored for 8 hours after the ingestion of the capsule and will continue to wear the data receiver and be contacted daily by phone at a pre determined time to enquire of any discomfort until the capsule is eliminated.

Who will see the information about you?
When you join the trial you will be given a unique identifier and thereafter all information will be filed with the code number, and stored in a locked filing cabinet accessed by the research team only. When information from all the participants have been pooled, and made anonymous, it will be used in presentations to academic societies, scientific publications. No names will be used, just the designated numbers. We will give you a summary of the findings of our research if you would like one.

All personal data will be destroyed at the end of the trial. Scientific data, filed on paper, will be shredded and electronic data will be deleted from our computer records and databases after 10 years. For the first 5 years it will be stored in a locked filing cupboard within a locked office. For the last 5 years it will be stored in a secure archive where all data is stored in boxes labelled by barcode only. It is accessible by nominated staff only who require pin numbers for ID.

If anything untoward is found in your tests you will be contacted by Professor Lentle, a medically qualified member of staff, who will inform you of the results and explain them to you. You will additionally be asked whether you would like the results to be given to your medical practitioner or sent directly to you.

Who is funding this research?
This study is funded by The Foundation for Research and Science Technology, The New Zealand Institute for Plant and Food Research and Blackcurrants New Zealand Incorporated, NZ.

Compensation for Injury
If physical injury results from your participation in this study, you should visit a treatment provider to make a claim to ACC as soon as possible. ACC cover and entitlements are not automatic and your claim will be assessed by ACC in accordance with the Accident Compensation Act 2001. If your claim is accepted, ACC must inform you of your entitlements, and must help you access those entitlements. Entitlements may include, but not be limited to, treatment costs, travel costs for rehabilitation, loss of earnings, and/or lump sum for permanent impairment. Compensation for mental trauma may also be included, but only if this is incurred as a result of physical injury.

If your ACC claim is not accepted you should immediately contact the researcher. The

researcher will initiate processes to ensure you receive compensation equivalent to that to which you would have been entitled had ACC accepted your claim from Massey University.

Will I get any financial compensation?

We will give you retail/petrol vouchers of $100 to compensate you for your inconvenience and time at each session that you come to the laboratory.

What are my rights?

You are under no obligation to accept this invitation. If you decide to participate, you have the right to:

- decline to answer any particular question;
- withdraw from the study at any time;
- ask any questions about the study at any time during participation;
- provide information on the understanding that your name will not be used unless you give permission to the researcher;
- be given access to a summary of the project findings when it is concluded.

If you are interested in participating please contact Ivana Sequeira who will be happy to discuss the project and answer your questions.

Ivana Sequeira
Institute of Food, Nutrition and Human Health
Massey University, Palmerston North
Tel/Text: 06 3569099 (81460) or 022 6751145
E mail: I.R.Sequeira@massey.ac.nz

Chris Booth
Institute of Food, Nutrition and Human Health
Massey University, Palmerston North
Tel/Text: 06 3569099 (5901) or 0800 0800 28
E mail: C.L.Booth@massey.ac.nz

This project has been reviewed and approved by the
Massey University Human Ethics Committee: Southern A, Application 12/42.
If you have any concerns about the conduct of this research, please contact
Dr Brian Finch, Chair, Massey University Human Ethics Committee: Southern A.
Telephone 06 350 5799 x 8717, email humanethicsoutha@massey.ac.nz.
