# Supplementary material for: Assessment of the Effect of Intestinal Permeability Probes (Lactulose And Mannitol) and Other Liquids on Digesta Residence Times in Various Segments of the Gut Determined by Wireless Motility Capsule: A Randomised Controlled Trial
Source: PLoS One. 2015 Dec 2;10(12):e0143690. doi: 10.1371/journal.pone.0143690 (PMC4667890; doi:10.1371/journal.pone.0143690)
Supplement: S4 File — (PDF) [file pone.0143690.s004.pdf]

MASSEY UNIVERSITY
TE KUNENGA KI PŪREHUROA
UNIVERSITY OF NEW ZEALAND

THE ENGINE OF THE NEW NEW ZEALAND

An assessment of the effect of food/neutraceuticals on transit time through various components of the human gut

AVOIDANCE OF PARTICULAR FOODS DURING THE COURSE OF THE STUDY

If you participate in this study you will be asked **to avoid the following a day before you are scheduled to come for your laboratory session** **as we are looking to assess the effect that a berry drink and a vitamin C drink (two of the five treatments that you will be randomised to) will have on your gut**. Hence you will have to avoid any fruits or vegetables (and their products) that are blue purple and yellow orange (as well as dark green leafy vegetables) in colour. Below is a guide to help you.

- Cantaloupe
- Citrus fruits and juices, such as orange and grapefruit
- Kiwi fruit
- Mango
- Papaya
- Pineapple
- Strawberries, raspberries, blueberries, cranberries
- Blackcurrants
- Any berries that are of the red/purple and blue varieties
- Any grapes that are of the black and red varieties
- Any juice that are of berry, grape, citrus flavour
- Any fruits and vegetables that have a red/purple/blue colouration (red cabbage, plums, aubergine/egg plant)
- Guava
- Watermelon
- Broccoli, Brussels sprouts, cauliflower
- Green and red peppers
- Spinach, cabbage, turnip greens, and other leafy greens
- Sweet and white potatoes
- Tomatoes and tomato juice
- Winter squash
- Black rice/ noodles
- Any juice that are of: berry or grape flavours
- Blackcurrant juice or cordial, cranberry
- Any conserves that contain berries (blackcurrant, blackberry boysenberry)
- Red wine

Some cereals and other foods and beverages are fortified with vitamin C. Fortified means a vitamin or mineral has been added to the food. Check the product labels to see how much vitamin C is in the product.

NOTE: Consumption of vitamin C-rich foods in their fresh, raw form is the best way to maximize vitamin C intake. The vitamin C content of food may be reduced by prolonged storage and by cooking. Hence, as the list contains many of the vegetables that are part of our daily diet, if you do have to eat it – reduce the amount you will eat and make sure that it is not raw i.e. in a salad or a quick stir fry.
Meat (chicken, pork, beef, lamb) and meat products, rice, noodles, cous cous, bread, can be eaten freely. Avocados, bananas, pears, asparagus are low in vitamin C. Also cucumbers, mushrooms, beans such as garbanzo beans, kidney beans, pinto beans, cooked corn are low in vitamin C.

Additionally, **dinner the previous night before you are scheduled to come to lab should not be a high fat meal.** We would also ask you to **limit the amount of salads/ fruits** that you will have with your meal. Kindly also take care to **avoid** any foods that could cause you GI irritation i.e. **spicy foods**.

Any questions you might have with regard to your food choices will be discussed with you by telephone or email or in person by Ivana Sequeira.
