# Supplementary material for: Assessment of the Effect of Intestinal Permeability Probes (Lactulose And Mannitol) and Other Liquids on Digesta Residence Times in Various Segments of the Gut Determined by Wireless Motility Capsule: A Randomised Controlled Trial
Source: PLoS One. 2015 Dec 2;10(12):e0143690. doi: 10.1371/journal.pone.0143690 (PMC4667890; doi:10.1371/journal.pone.0143690)
Supplement: S5 File — (PDF) [file pone.0143690.s005.pdf]

SmartPill
The Measure of GI Health

SmartPill Capsule

Important Patient Information and Instructions

1. The Evening Before the Test

- You must fast overnight before your test. Do not eat or drink anything after 10.00pmthe night before the test.
- Refrain from alcohol consumption 24 hours before the test and for the duration of the test.
- If you have started taking medications kindly let us know.

2. The Day of the Test

- Do not eat or drink anything before coming to the HNU, IFNHH. The SmartPill test will include a meal that will be provided to you at the end of the experimental session.
- You should arrive at the HNU, IFNHH at **8.00am**

3. For the Duration of the Test

- You must wear the data receiver on your body at all times for the duration of the test except when you bathe or shower. The data receiver can be worn on a lanyard around the neck or on a belt clip.

  CAUTION

  Do not bathe while wearing the data receiver – when you shower or bathe, you must remove the data receiver and place it as near to the shower or bathtub as possible.

  CAUTION

  Do not use the lanyard when sleeping.

- Six (6) hours after capsule ingestion we will provide you with a meal and thereafter you may resume your normal diet.
- Refrain from alcohol consumption until after the SmartPill capsule is passed.
- Refrain from using laxatives, bowel cathartics, anti-diarrhea medications, and other medications that affect motility until after the SmartPill capsule is passed.
- Avoid vigorous exercise such as sit-ups, abdominal crunches, and prolonged aerobic activity (greater than 15 minutes) until after the SmartPill capsule is passed.
- During the test, wait two (2) minutes in the lavatory before flushing the toilet after each bowel movement.

Contact Information:
Ivana Sequeira
Institute of Food, Nutrition and Human Health
Massey University, Palmerston North
Tel/Text: 06 3569099 (81469) or 022 6751145
E mail: I.R.Sequeira@massey.ac.nz
