# Supplementary material for: Assessment of the Effect of Intestinal Permeability Probes (Lactulose And Mannitol) and Other Liquids on Digesta Residence Times in Various Segments of the Gut Determined by Wireless Motility Capsule: A Randomised Controlled Trial
Source: PLoS One. 2015 Dec 2;10(12):e0143690. doi: 10.1371/journal.pone.0143690 (PMC4667890; doi:10.1371/journal.pone.0143690)
Supplement: S6 File — (PDF) [file pone.0143690.s006.pdf]

SUBJECT NAME: ______________________________________

This person has swallowed a monitoring device called the SmartPill®. As this Capsule contains metal this person should not be allowed to undergo MRI scanning. In case of any medical emergencies or other difficulties day or night kindly contact:

Ivana Sequeira
Institute of Food, Nutrition and Human Health
Massey University, Palmerston North
Tel/Text: 06 3569099 (81469) or 022 6751145
E mail: I.R.Sequeira@massey.ac.nz

This project has been reviewed and approved by the
Massey University Human Ethics Committee: Southern A, Application 12/42.
If you have any concerns about the conduct of this research, please contact
Dr Brian Finch, Chair, Massey University Human Ethics Committee: Southern A.
Telephone 06 350 5799 x 8717, email humanethicsoutha@massey.ac.nz.
