# Supplementary material for: Assessment of the Effect of Intestinal Permeability Probes (Lactulose And Mannitol) and Other Liquids on Digesta Residence Times in Various Segments of the Gut Determined by Wireless Motility Capsule: A Randomised Controlled Trial
Source: PLoS One. 2015 Dec 2;10(12):e0143690. doi: 10.1371/journal.pone.0143690 (PMC4667890; doi:10.1371/journal.pone.0143690)
Supplement: S7 File — (PDF) [file pone.0143690.s007.pdf]

Standard operating procedure (SOP) for Collection of Faecal Samples

We require you to collect you stool samples as it is important that we monitor the passage of the capsule from your GI tract. This procedure had to be adhered to every time you have a bowel movement.

You will be provided with the following:

- Plastic bowl
- Gloves
- Sealable plastic bags
- Large Biohazard bag

First open the pack and then place the plastic bowl in the middle of the toilet bowl (this sits well in the bowl so that all faeces will be collected comfortably).

Label the bag with the date and the time and then place the sealable bag on top of the plastic bowel and draw the ends of the bag over the toilet seat.

DO NOT worry if urine is mixed with the fecal matter.

Once you have finished let the stool remain in the bag for 3 minutes, this time allowing the data receiver to detect any temperature changes so that it can record when the SmartPill® has been passed out of your body.

You will also be asked to visually confirm the evacuation of the capsule which you must record on your diary.

Wear the gloves and seal the small plastic bag provided and place it in the biohazard bag provided for storage.

The biohazard bag should be placed in a convenient cool place e.g. in a garage, garden shed pending collection/drop off by you.

All bags that have been returned to us will then be frozen until they are collected for disposal by incineration by Interwaste.

NOTE: We will demonstrate how you can place the bowl and the plastic bags onto the toilet bowl when you come to the laboratory so that you can see what is expected of you. Ivana will be happy to answer any questions and discuss any problems etc.
