# Supplementary material for: Assessment of the Effect of Intestinal Permeability Probes (Lactulose And Mannitol) and Other Liquids on Digesta Residence Times in Various Segments of the Gut Determined by Wireless Motility Capsule: A Randomised Controlled Trial
Source: PLoS One. 2015 Dec 2;10(12):e0143690. doi: 10.1371/journal.pone.0143690 (PMC4667890; doi:10.1371/journal.pone.0143690)
Supplement: S8 File — (DOCX) [file pone.0143690.s008.docx]

DATA SET UNDERLYING FINDINGS OF THE STUDY

1. Raw data of the derived transit times from pH and pressure recordings from the SmartPill for each segment for each participant following each treatment

| **SUBJECT** | **VISIT** | **TREATMENT** | **GET** | **SBTT** | **LBTT** |
| --- | --- | --- | --- | --- | --- |
| SP01 | 1 | Placebo | 1.37 | 5.22 | 80.55 |
| SP01 | 2 | Lac Man | 1.46 | 4.72 | 114.96 |
| SP01 | 3 | Aspirin | 1.16 | 5.03 | 68.36 |
| SP01 | 4 | Blackcurrant | 2.13 | 5.10 | 50.11 |
| SP01 | 5 | Ascorbic acid | 1.22 | 6.01 | 79.12 |
| SP02 | 1 | Blackcurrant | 1.35 | 5.08 | 73.66 |
| SP02 | 2 | Placebo | 0.54 | 4.49 | 72.47 |
| SP02 | 3 | Aspirin | 1.20 | 4.08 | 43.12 |
| SP02 | 4 | Ascorbic acid | 0.54 | 4.87 | 28.64 |
| SP02 | 5 | Lac Man | 1.34 | 4.99 | 67.67 |
| SP03 | 1 | Lac Man | 1.58 | 3.45 | 25.36 |
| SP03 | 2 | Aspirin | 6.20 | 2.89 | 72.45 |
| SP03 | 3 | Placebo | 1.38 | 5.13 | 24.97 |
| SP03 | 4 | Ascorbic acid | 1.26 | 5.86 | 75.07 |
| SP03 | 5 | Blackcurrant | 4.19 | 3.20 | 24.74 |
| SP04 | 1 | Placebo | 1.51 | 3.52 | 76.50 |
| SP04 | 2 | Ascorbic acid | 1.40 | 4.69 | 3.26 |
| SP04 | 3 | Blackcurrant | 3.16 | 7.94 | 45.30 |
| SP04 | 4 | Aspirin | 1.25 | 4.97 | 49.18 |
| SP04 | 5 | Lac Man | 2.02 | 5.24 | 43.77 |
| SP05 | 1 | Lac Man | 3.21 | 7.14 | 16.89 |
| SP05 | 2 | Ascorbic acid | 1.55 | 4.70 | 27.93 |
| SP05 | 3 | Blackcurrant | 1.36 | 4.78 | 19.37 |
| SP05 | 4 | Placebo | 2.25 | 4.78 | 22.51 |
| SP05 | 5 | Aspirin | 1.42 | 4.04 | 19.89 |
| SP06 | 1 | Aspirin | 1.24 | 3.17 | 93.84 |
| SP06 | 2 | Ascorbic acid | 0.43 | 3.76 | 116.92 |
| SP06 | 3 | Lac Man | 1.14 | 2.29 | 92.97 |
| SP06 | 4 | Placebo | 1.12 | 3.14 | 57.04 |
| SP06 | 5 | Blackcurrant | 1.58 | 4.60 | 20.98 |

*GET = gastric emptying time; SBTT = small bowel emptying time; LBTT = large bowel / colonic emptying time

Data was transformed for analysis as stated in text.

1. Mean quartile values of small intestinal pH for each participant following each treatment

| **SUBJECT** | **TREATMENT** | **Quartile 1** | **Quartile 2** | **Quartile 3** | **Quartile 4** |
| --- | --- | --- | --- | --- | --- |
| 1 | Aspirin | 5.84 | 6.95 | 7.37 | 7.34 |
| 1 | Placebo | 5.89 | 7.05 | 7.8 | 7.5 |
| 1 | Lac Man | 1.33 | 6.38 | 7.55 | 7.61 |
| 1 | Blackcurrant | 6.22 | 6.99 | 7.29 | 7.24 |
| 1 | Ascorbic acid | 6.06 | 6.73 | 7.46 | 7.49 |
| 2 | Aspirin | 5.01 | 6.36 | 7.19 | 7.39 |
| 2 | Placebo | 4.89 | 6.46 | 7.47 | 7.6 |
| 2 | Lac Man | 4.997 | 6.92 | 7.55 | 7.61 |
| 2 | Blackcurrant | 5.82 | 6.99 | 7.31 | 7.07 |
| 2 | Ascorbic acid | 4.63 | 6.13 | 7.19 | 7.32 |
| 3 | Aspirin | 5.57 | 6.67 | 7.07 | 7.46 |
| 3 | Placebo | 5.49 | 6.42 | 7.12 | 7.52 |
| 3 | Lac Man | 4.45 | 6.52 | 7.23 | 7.21 |
| 3 | Blackcurrant | 5.22 | 5.91 | 6.79 | 6.77 |
| 3 | Ascorbic acid | 6.07 | 6.38 | 7.34 | 7.39 |
| 4 | Aspirin | 5.24 | 6.81 | 7.55 | 7.74 |
| 4 | Placebo | 4.15 | 6.12 | 7.18 | 7.31 |
| 4 | Lac Man | 5.4 | 6.36 | 7.41 | 7.54 |
| 4 | Blackcurrant | 5.5 | 7.09 | 7.17 | 7.3 |
| 4 | Ascorbic acid | 4.3 | 6.19 | 7.08 | 7.3 |
| 5 | Aspirin | 4.31 | 7.05 | 7.45 | 7.46 |
| 5 | Placebo | 5.62 | 7.02 | 7.32 | 7.56 |
| 5 | Lac Man | 5.43 | 7.18 | 7.48 | 7.6 |
| 5 | Blackcurrant | 4.94 | 7.01 | 7.27 | 7.36 |
| 5 | Ascorbic acid | 4.11 | 6.8 | 7.38 | 7.43 |
| 6 | Aspirin | 5.14 | 6.77 | 7.38 | 7.52 |
| 6 | Placebo | 5.67 | 6.6 | 7.21 | 7.41 |
| 6 | Lac Man | 5.32 | 6.22 | 7.32 | 7.52 |
| 6 | Blackcurrant | 4.52 | 7.08 | 7.31 | 7.33 |
| 6 | Ascorbic acid | 4.57 | 6 | 6.8 | 7.05 |

Data was transformed for analysis as stated in text.

1. Mean proximal and distal colonic pH for each participant following each treatment

| **SUBJECT** | **TREATMENT** | **PROXIMAL** | **DISTAL** |
| --- | --- | --- | --- |
| 1 | Aspirin | 5.4690439 | 7.8874724 |
| 2 | Aspirin | 5.6336568 | 6.9446366 |
| 3 | Aspirin | 6.7933967 | 7.2140231 |
| 4 | Aspirin | 7.862362 | 7.0823085 |
| 5 | Aspirin | 6.4252407 | 7.2839137 |
| 6 | Aspirin | 5.8838064 | 7.4829133 |
| 1 | Placebo | 5.9804504 | 7.3989002 |
| 2 | Placebo | 5.9516956 | 7.3064418 |
| 3 | Placebo | 6.8609234 | 7.1607403 |
| 4 | Placebo | 6.9546517 | 7.4201793 |
| 5 | Placebo | 6.1278467 | 7.0948335 |
| 6 | Placebo | 5.466122 | 6.8287303 |
| 1 | Lac Man | 5.7066114 | 8.0566618 |
| 2 | Lac Man | 6.162787 | 7.585521 |
| 3 | Lac Man | 6.1653434 | 7.3178083 |
| 4 | Lac Man | 6.6829414 | 6.4608963 |
| 5 | Lac Man | 5.794426 | 6.7664823 |
| 6 | Lac Man | 6.2091044 | 7.9983132 |
| 1 | Blackcurrant | 5.4284212 | 7.4099281 |
| 2 | Blackcurrant | 5.6843197 | 7.1014042 |
| 3 | Blackcurrant | 6.7115575 | 6.9043753 |
| 4 | Blackcurrant | 6.8832764 | 7.1342125 |
| 5 | Blackcurrant | 6.8239249 | 7.4013224 |
| 6 | Blackcurrant | 5.6531805 | 6.5268482 |
| 1 | Ascorbic acid | 5.6068457 | 7.9448106 |
| 2 | Ascorbic acid | 5.6966766 | 6.5301082 |
| 3 | Ascorbic acid | 6.409084 | 7.1194364 |
| 4 | Ascorbic acid | 5.7915263 | 6.2878735 |
| 5 | Ascorbic acid | 6.6276205 | 7.064453 |
| 6 | Ascorbic acid | 5.7511006 | 8.6249781 |
